# pr2‐Wormifier: A Bioinformatics Pipeline to Create Custom Reference Databases for Improved Metabarcoding of Marine Protists

**DOI:** 10.1111/1755-0998.70168

**Published:** 2026-06-23

**Authors:** Stefanie Knell, Juliane Romahn, Miklós Bálint

**Affiliations:** ^1^ Justus Liebig University Giessen Giessen Germany; ^2^ Senckenberg Biodiversity and Climate Research Centre Frankfurt am Main Germany; ^3^ Centre for Translational Biodiversity Genomics Frankfurt am Main Germany

**Keywords:** 18S rRNA, ciliate, environmental DNA, sedaDNA, taxonomic affiliation, WoRMs

## Abstract

Metabarcoding of environmental and ancient environmental DNA (eDNA and sedaDNA) is a powerful approach for studying and monitoring marine communities. However, its effectiveness is limited by the availability of comprehensive and well‐curated reference databases, particularly for protists. Here, we introduce pr2‐wormifier, a bioinformatics pipeline designed to create customized and improved reference databases for 18S rRNA‐based metabarcoding. This pipeline integrates sequences from PR^2^ and NCBI with taxonomic information from the World Register of Marine Species (WoRMS) and AlgaeBase, allowing for refined taxonomic assignments at the genus and species levels. pr2‐wormifier enables users to tailor reference databases to specific taxonomic groups or geographic regions, enhancing the resolution and accuracy of biodiversity assessments. We benchmarked the pipeline using a sedimentary ancient DNA dataset from the Baltic Sea, focusing on marine protists, especially ciliates and dinoflagellates. The customized database generated by pr2‐wormifier identified more sequences at the genus and species levels than PR^2^ alone, while maintaining taxonomic consistency and quality. Our results demonstrate that pr2‐wormifier addresses common limitations of existing databases, such as low taxonomic resolution and missing taxa and facilitates more reliable classification in metabarcoding studies. By enabling the creation of locally relevant, taxonomically curated databases, pr2‐wormifier offers a flexible and scalable solution for improving the identification of protists in environmental and paleoenvironmental research.

## 1 Introduction

The taxonomic identification of species based on short, standardized fragments of DNA, known as DNA barcoding, has established itself as an important method for biodiversity assessment (Hebert et al. 2003). The availability of high‐throughput sequencing enables metabarcoding, the identification of multiple species from bulk samples or environmental samples containing DNA (Taberlet et al. 2012). Metabarcoding can bring advances for the assessment of communities relevant for many research fields such as ecology, conservation biology, and invasion biology, as well as biomonitoring (Deiner et al. 2017).

Metabarcoding of environmental DNA (eDNA) has many advantages for surveying aquatic communities such as non‐invasiveness, low monitoring effort, and high species detectability, especially for species and in places that are hard to monitor through traditional methods (Valentini et al. 2016). Additionally, metabarcoding of sedimentary ancient DNA (sedaDNA) established itself as a method to investigate past biodiversity (Nguyen et al. 2023).

These new methods can be particularly beneficial for the study of protists (Burki et al. 2021), a paraphyletic group of generally unicellular eukaryotes (Adl 2005). Protists are highly diverse, both taxonomically and functionally, and as such an important part of marine communities (Corliss 2002). Phototrophic protists, such as diatoms, play a crucial role in the primary production of marine ecosystems (Sarthou et al. 2005), whereas heterotrophic protists like ciliates can link primary producers and larger‐size zooplankton in the food web (Calbet and Saiz 2005). Large‐scale metabarcoding studies of marine protists show that the existing diversity greatly exceeds what has been described morphologically, especially for rare and small protist taxa (de Vargas et al. 2015). Metabarcoding will remain an important approach for understanding not only protist diversity but also their distribution, community structure, ecosystem functions, and trophic interactions (Santoferrara et al. 2020).

The small subunit ribosomal RNA gene (18S) consists of both highly conserved and variable regions (Nelles et al. 1984). This allows us to design primers that amplify a broad range of eukaryotic diversity (Hadziavdic et al. 2014). While other barcoding markers allow for higher taxonomic resolution for certain groups within protists (Pawlowski et al. 2012), the 18S region is the most widely used due to its ability to target protist diversity as a whole (Capo et al. 2015; Santoferrara et al. 2020; Zimmermann et al. 2024).

The ability and reliability of metabarcoding to describe full communities is limited by the availability of comprehensive and well‐curated reference databases (Keck et al. 2023). Rare and understudied taxa are often missing in reference databases and therefore cannot be detected (Kermarrec et al. 2014; Sinniger et al. 2016). Incomplete reference databases may also lead to false positive assignments to related taxa, especially for methods that are not tree‐based such as BLAST (Ross et al. 2008; Schenekar et al. 2020). To account for intraspecific variation, it is also desirable to have sequences of multiple specimens in the database that ideally cover the geographical range of the species (Brodin et al. 2013; Keck et al. 2023). Additionally, sequences with wrong or low taxonomic resolution can obstruct taxonomic assignments by both hindering assignments or creating false positives (Meiklejohn et al. 2019; Viard et al. 2019). Erroneous sequence entries are not uncommon in public databases and are often the result of misidentification, contamination or sample confusion (Cheng et al. 2023; Keck et al. 2023). Furthermore, taxonomically unresolved groups and the discovery of new lineages are a challenge for creating reference databases (del Campo et al. 2018).

NCBI GenBank (Benson et al. 2000) as part of the International Nucleotide Sequence Database Collaboration is the most comprehensive collection of genetic sequences. It is widely used for metabarcoding studies but is problematic for many taxonomic groups due to issues in sequence quality, misidentification and annotations of groups (Bidartondo 2008; Harris 2003). To address these issues, curated, specialized reference databases have been established. Of those, the SILVA ribosomal RNA gene database (Quast et al. 2013) and the Protist Ribosomal Reference database (PR^2^; Guillou et al. 2013) are the most commonly used for protist identification. The SILVA database aims to provide a comprehensive, quality checked collection of ribosomal sequences from microbes of all three domains of life. PR^2^ is primarily a reference database for protists. Apart from protists, PR^2^ also contains a certain amount of sequences from other groups such as metazoa, land plants and macrosporic fungi. This allows to distinguish these groups and to prevent false positive assignments to protists when using broad range eukaryotic primers. While using PR^2^ as a reference database has many advantages for metabarcoding of protists, the database also has some limitations. Since PR^2^ is updated periodically through new releases, new public 18S sequences might be incorporated with a delay. Additionally, the database contains various sequences with low taxonomic resolution. Furthermore, since certain taxonomic groups are subdivided (e.g., Tintinnopsis_01 bis Tintinnopsis_13), new species cannot be easily added to the database without extensive taxonomic knowledge. Given these limitations, we sought to prepare a pipeline which can create enhanced, customized reference databases to improve the taxonomic accuracy, completeness and applicability of metabarcoding for protists.

We implemented a bioinformatics pipeline that amends the PR^2^ reference sequence database in an automated way. The aim is to optimize the database for improved taxonomic assignment, especially on species and genus level, for a set of species that are of interest to the user. This includes replacing the PR^2^ taxonomy by one created based on information from the World Register of Marine Species (WoRMS; Ahyong et al. 2025) and AlgaeBase (Guiry and Guiry 2023) as well as adding additional sequences from NCBI. We then benchmarked the pipeline by building a reference database for Baltic marine eukaryotes and testing its ability to identify ciliates and dinoflagellates (Dinophyceae) for two different environmental DNA (eDNA) metabarcoding datasets. We chose ciliates and dinoflagellates as examples because they are commonly studied using 18S metabarcoding (Abraham et al. 2024; Ganser et al. 2021; Majaneva et al. 2024). Both are important in Baltic food webs (Fridolfsson et al. 2023; Mironova et al. 2009; Setälä and Kivi 2003), particularly for dinoflagellates, which are prominent during the spring bloom (Lundsør et al. 2022; Wasmund et al. 2017).

## 2 Materials and Methods

### 2.1 Implementation of the Pipeline

**FIGURE 1:**
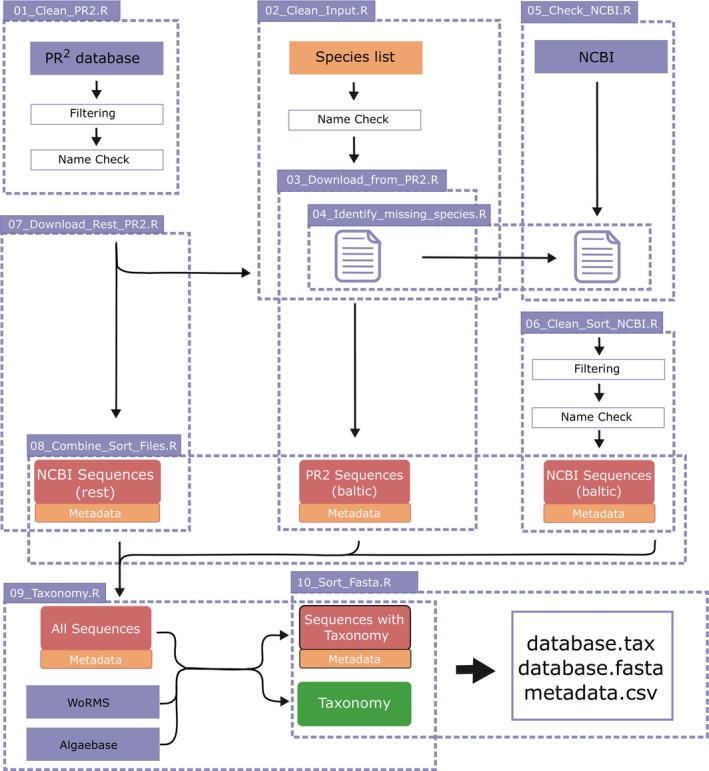
Schematic overview over the bioinformatics pipeline to build custom reference databases. The species list provided by the user is represented in yellow, sources for sequences in blue and sources for taxonomy in green. The dotted boxes indicate which part of the pipeline is to be found in which script, highlighting the main data handled in those parts.

The pipeline consists of 10 scripts, nine of them written in R statistics (Figure 1). It reduces the PR^2^ database by removing sequences with low taxonomic resolution and sequences of cell organelles. The pipeline then searches for the taxonomy of all remaining species in WoRMS and AlgaeBase and replaces species names with the currently accepted ones if available. The remaining database is then compared with a list of species of interest provided by the user. For those species, additional sequences are added from NCBI if available. In the final step, the taxonomy provided by PR^2^ is replaced by a new taxonomy based on WoRMS and AlgaeBase for all genera that already exist within these databases. Taxa without WoRMS or Algaebase taxonomy are removed (reasoning below).

All R scripts were written in R version 4.5.1. The following R packages were used for data wrangling are: *dplyr* (v1.2.1; Wickham et al. 2023), *plyr* (v.1.8.9; Wickham 2011), *tidyr* (v1.3.2; Wickham et al. 2024), *stringr* (v1.6.0; Wickham 2025), *stringi* (v1.8.7; Gagolewski 2022), *purrr* (v1.2.2; Wickham and Henry 2026); for sequence handling: *pr2database* (v5.1.2; Vaulot 2026), *seqinr* (v4.2.36; Charif and Lobry 2007), *phylotools* (v0.2.2; Zhang 2024) *Biostrings* (v2.78.0; Pagès et al. 2025); for taxonomy databases: *worrms* (v0.4.3; Chamberlain and Vanhoorne 2023), *taxonomizr* (v0.11.1; Sherrill‐Mix 2025); for NCBI access: *entrez* (v1.2.3; Winter 2017); for API to access Algaebase *jsonlite* (v2.0.0; Cooley 2022), *curl* (v6.2.2; Ooms 2025).

The scripts 0*1_Clean_PR2*, *02_Clean_Input*, 0*5_Check_NCBI* and 0*9_Taxonomy* were run between 13.05.2025 and 23.05.2025 and accessed the version of the databases WoRMS, AlgaeBase and NCBI that were available online to that date. Sequences were downloaded from NCBI on 15.05.2025 via 0*5_Check_NCBI*. The current version of the PR^2^ reference database accessed through the R package was 5.1.1 released on 10.10.25.

### 2.2 Cleaning the PR^2^ Database and Preparing Species Lists

The first two scripts of the pipeline, 0*1_Clean_PR2* and 0*2_Clean_Input*, pre‐organize both the existing PR^2^ database and the list of species of interest provided by the user. The PR^2^ reference sequence database consists of sequences of the gene coding for the small ribosomal subunit (18S). This includes sequences of homologous genes from different cell organelles, in particular mitochondria, nucleomorphs, plastids and apicoplasts, that are labelled with a colon and a four‐letter abbreviation throughout the taxonomy (e.g., *Fungi: mito*). These organelle sequences are filtered out since they are not useful in the classification of nuclear 18S sequences. Additionally, sequences are removed that are not identified to the genus or species level, as sequences with low taxonomic resolution can hinder classification for taxa where sequences with more complete taxonomy are available. If multiple reference sequences of the same species or amplicon exist in the database, assignment with some of the available tools (e.g., mothur, Schloss et al. 2009) is only possible down to the lowest taxonomic rank at which all reference sequences share the same taxonomy. We choose to keep sequences without species information (e.g., *Uronema* sp.), even though they might obstruct species‐level assignment in some cases, as removing them would considerably reduce the size and comprehensiveness of the reference database.

To enable accurate search within the database, all species names should be strictly in the binomial format of genus and specific epithet. Therefore, all other elements are removed from species names, such as the label *Candidatus* leading up to certain bacterial genera, numbers used within PR^2^ taxonomy to divide certain taxa into smaller groups or number and letter combinations to indicate stems. For some species, small formatting errors are corrected. Bacterial species with the same name as eukaryotic species are removed (e.g., *Candidatus_Chlorothrix_sp*. vs. the algae *Chlorothrix_sp*.).

Many species names have multiple synonyms that are used throughout the literature. To avoid mismatches and to be able to accurately determine which species are in the database, the most recent accepted name should be used. To achieve that, both the species in the database and the species in the list provided by the user are searched for in WoRMS using the Taxamatch fuzzy matching algorithm as implemented in the R package ‘worrms’ (Chamberlain and Vanhoorne 2023). For species where a WoRMS record can be unambiguously assigned, the accepted WoRMS name is used from here on. Species without a WoRMS record are kept with the cleaned version of the name they had in PR^2^ or the list provided by the user.

Phytoplankton species are additionally searched in AlgaeBase. All synonyms known to WoRMS are added to the list of species provided by the user to enable comprehensive literature research when interpreting results.

### 2.3 Searching for Sequences of Species of Interest

The script 0*3_Download_from_PR2* searches for all species of interest provided by the user in the cleaned PR^2^ reference database as prepared in script one. Available sequences from these species and species of the same genus are downloaded into separate files. A metadata file is created which contains the (i) PR^2^ accession number, (ii) GenBank accession number, (iii) AphiaID (if available), (iv) accepted name of species or higher level taxa from WoRMS, (v) accepted name from AlgaeBase, (vi) the cleaned version of the name as prepared in script *1_Clean_PR2*, (vii) the original name from PR^2^ database, (viii) information about the sequence length, (ix) details of the database and (x) the replicate number of the sequence. The AphiaID is a unique identifier that exists for every taxonomic name within WoRMS, provided by Aphia, the infrastructure WoRMS is based on (Vandepitte et al. 2015). It can be used to extract trait information from WoRMS.

The script 0*4_Identify_missing_species* prepares two lists based on the user's species of interest. The first one contains all species for which no sequences could be downloaded from PR^2^. The second one contains species that are already present in the database. These lists are the basis for the R script *5_Check_NCBI* to search for and add additional sequences from the NCBI nucleotide database (Geer et al. 2010). The search query consists of a species or genus name in the field organism, in combination with the search terms ‘18S’ or ‘small’. To reduce the number of false search results, only entries are considered that are not labelled as chloroplast or mitochondrion. Search results are limited to sequences with a length between 350 and 5000 bp. The script first searches for species that are still missing from the database. In a second step, sequences are added from additional species of genera that are of interest. This improves the taxonomic assignment of species for which no reference is available. These species cannot be identified through metabarcoding, but having sequences of the same genus in the database enables classification on genus level. For genera that comprise more than one species it is also desirable to have multiple sequences from different species in the database. This reduces the risk of false‐positive species‐level assignments.

The following three scripts 0*6_Clean_Sort_NCBI_Downloads*, 0*7_Download_Rest_PR2* and 0*8_Combine_Sort_Files* filter all NCBI downloads and combine them with the rest of the PR^2^ database. To avoid duplicates in the database, the downloaded sequences are filtered by accession number to remove those that already exist in PR^2^. Additionally, all sequences are filtered out that are not nuclear 18S sequences or contain only small fractions of the 18S region. Similarly to scripts 0*1_Clean_PR2* and *2_Clean_Input*, the species names are cleaned to be in binomial format and searched in WoRMS to retrieve their accepted name. Each sequence is written into a separate file and gets a replicate number.

### 2.4 Taxonomy

The nine‐level taxonomy built in script 09_Taxonomy is retrieved from WoRMS and AlgaeBase for all sequences in the database, regardless of whether these sequences originate from PR^2^ or NCBI. This includes updating genus and species names to their currently accepted nomenclature. The following ranks are used: kingdom, phylum, subphylum, class, subclass, order, family, genus, and species. If one of these ranks is not available, the rank above is used with an X added (e.g., Chlorophycea_X). For entries that already have an AphiaID, taxonomy is directly retrieved from WoRMS. This taxonomy is also used for sequences of the same genus that do not have an AphiaID. All remaining taxa are searched for in WoRMS on genus level.

If multiple WoRMS records are returned for the same name (this can occur when the same name has been applied to unrelated taxonomic groups, for example, the ciliate *Uronema* and the green alga *Uronema*), the correct record is identified using the taxonomic context available in PR^2^ or the user‐defined species list. This same approach is applied to remove incorrectly downloaded NCBI sequences that share the same genus name but belong to a different lineage, using higher taxonomic ranks (e.g., class or phylum) as a discriminating criterion. In some cases, multiple records may be retained which often have the same taxonomy. In those cases, we chose the record labelled ‘accepted’. If this is true for multiple records, we chose the last edited record. If the records were edited at the same time, we chose the one with the highest AphiaID. Sequences for which no taxonomy can be found in WoRMS are removed from the database. The last script *10_Sort_Fasta* creates the final fasta file of the reference sequences containing only sequences for which taxonomy exists.

## 3 Benchmarking

### 3.1 Optimizing the Database for Baltic Marine Eukaryotes

We benchmarked the pipeline by creating an optimized database for eukaryotic species of the Baltic Sea. To get a comprehensive species list for the Baltic Sea we combined the Checklist 2.0 of Baltic Sea macrospecies (HELCOM 2020) and the Checklist of Baltic Sea Phytoplankton Species (HELCOM 2004), both published by the Helsinki Commission (HELCOM), with the species list included in the Zooplankton of the Open Baltic Sea: Atlas (Telesh et al. 2008). This resulted in a total of 6264 species and 2596 genera for which additional sequences were searched for in NCBI.

To test the new database, we used two metabarcoding datasets generated from environmental DNA from the Baltic Sea. The first dataset published by Nguyen et al. (2026) included recent DNA extracted from seawater and surface sediments in the Gulf of Gdańsk, the South Eastern Baltic Sea. The second dataset contains sedimentary ancient DNA from the Gulf of Finland, the Eastern Gotland Basin and Landsort, published in Romahn et al. (2025). Bioinformatic processing and bioinformatic cleaning, including more detailed sample information, follow in separate parts. The following steps, including species classification and the statistical calculation, were handled the same way.

### 3.2 Metabarcoding eDNA Dataset From Seawater and Surface Sediments (TAReuk)

This dataset includes two journeys of water samples from the entire water column and corresponding surface samples of the Gulf of Gdańsk, the South‐Eastern Baltic Sea (Nguyen et al. 2026). This dataset is a eukaryotic metabarcoding dataset consisting of ~387 bp amplicons of the 18S rRNA V4 region amplified with TAReuk454FWD1/TAReukREV3 (Forward: 5′‐CCAGCASCYGCGGTAATTCC‐3′; Reverse: 5′‐ACTTTCGTTCTTGATYRA‐3′; Stoeck et al. 2010). The dataset contains 24 samples in total, each with 3 PCR replicates, along with several sampling, extraction, PCR negative controls and tag‐jump controls. The sequencing data are accessible on NCBI under the BioProject number PRJNA1320821.

The bioinformatic processing involved ObiTools4 (v 4.0.4; Boyer et al. 2016) for read merging with a minimum overlap of 10 bp and alignment scores of 0.8, dereplication and prefiltering as recommended by the ObiTools Manual. We removed ASVs with counts below 10, as described by Nguyen et al. (2026). In addition, we removed ASVs with fewer than 56 reads based on ASV frequency plots (Taberlet et al. 2018). We subtracted the maximum read count for each ASV in the controls from the replicates. Successful data cleaning was verified by assessing community composition using nonmetric multidimensional scaling (NMDS) with the ‘vegan’ package (Oksanen et al. 2024) and a seed number of 25. We finally averaged the replicate reads of each ASV in each sample.

### 3.3 Metabarcoding Sedimentary Ancient DNA Data Set (Euka02)

This dataset includes sedimentary ancient DNA (sedaDNA) data from sediment cores, spanning the period between 1800 and 2021 ce. The dataset is an eukaryotic metabarcoding dataset that targets ~158 bp amplicons of the 18S rRNA V4 region, amplified with the Euka02 primer (Forward: 5′‐TTTGTCTGSTTAATTSCG‐3′, Reverse: 5′‐CACAGACCTGTTATTGC‐3′; Guardiola et al. 2015). The dataset contains 110 samples in total, each with 4 PCR replicates, along with several extraction and PCR negative controls as tagjump controls. A detailed description regarding sampling, dating, laboratory procedures and bioinformatic cleaning of the metabarcoding data can be found in (Schmidt et al. 2024) and (Romahn et al. 2025). The sequencing data are available at the European Nucleotide Archive of the European Bioinformatics Institute (EMBL‐EBI) under accession PRJEB85715.

The bioinformatic processing involved ObiTools4 (v 4.0.4; Boyer et al. 2016) for read merging, demultiplexing, dereplication and prefiltering as recommended by the ObiTools Manual. Additionally, sequences shorter than 80 bp and longer than 300 bp, as well as those with a count below 10, were discarded. Amplicon sequence variants (ASVs) with a total read count of less than 85 were considered low‐frequency and excluded. Replicates with fewer than 100,000 reads were removed due to likely PCR failure. The maximum read number of each ASV in negative controls was calculated and subtracted from each sample replicate. Samples with only 2 remaining replicates were discarded. Successful data cleaning was verified by assessing community composition using nonmetric multidimensional scaling (NMDS) with the ‘vegan’ package (Oksanen et al. 2024) and a seed number of 25. Finally, the read count of each ASV was normalized to 500 μg sediment.

### 3.4 Species Classification of Both Datasets

To benchmark the individual components of pr2‐wormifier, the species classification of the sedaDNA metabarcoding data was done with four different versions of the reference database. Two of them are the original PR^2^ database (v1) and the final database created by the pipeline as described above (v4). Additionally, two in‐between versions of the PR^2^ database are used that contain only the PR^2^ sequences that remain in the database throughout the pipeline, once with their PR^2^ taxonomy (v2) and once with their new WoRMS taxonomy (v3). This allows us to examine how changes to the database, removing sequences, changing taxonomy and adding sequences impact the results.

To improve species classification, we performed *in silico* PCR on all four versions of the database, using the function *insilico_pcr* from the software CRABS (v0.1; Jeunen et al. 2023). For the maximum number of allowed errors at the primer binding site, the default value of 4.5 was kept. This reduced the database to the specific region that is amplified by the primers. In this step, partial 18S sequences were sorted out that do not cover the region of the barcode and might otherwise influence species classification. The software also detects primer binding sites in sequences that are in the database as reverse complements.

The species classification was done using the software mothur (Schloss et al. 2009) using the classify.seqs() command with a bootstrap confidence threshold of 80% and the naive Bayesian RDP classifier algorithm (Wang et al. 2007).

### 3.5 Comparison of Databases Based on Both Datasets

We compared the results of the species classification done with the four different database versions, as defined above. The PR^2^ taxonomy and the one we built based on WoRMS and AlgaeBase differ in the ranks and the taxonomic groups they use, although they both contain nine taxonomic levels. Therefore, we limited the comparison on taxonomic assignment to the genus and species level, which are the ranks that are identical between the PR^2^ and WoRMS‐AlgaeBase taxonomies. The comparison was conducted in R with the following additional R packages: *ggplot2* (v4.0.2; Kassambara 2023) *tibble* (v3.3.1; Müller and Wickham 2023), *gapminder* (v1.0.1; Bryan 2023), *treemapify* (v2.6.0; Wilkins 2023), *ggpubr* (v0.6.1; Kassambara 2023), *ghibli* (v0.3.4; Henderson 2024), *openxlsx* (v4.2.8.1; Schauberger and Walker 2025), *legendry* (v0.2.4; van den Brand 2025b), *ggh4x (*v0.3.1; van den Brand 2025a).

First, we briefly compared the total number of species and the number of genus assignments. We then focused on the taxonomic group Ciliophora and Dinophyceae (Dinoflagellates). Ciliophora occurs as a phylum in WoRMS‐Algaebase taxonomy and as a class in the PR^2^ taxonomy. Dinophyceae occurs as a Subclass in WoRMS‐Algaebase taxonomy and as a class in the PR^2^ taxonomy. For both taxa, we compared the number of ASVs that could be assigned to family, genus and species level, the corresponding number of reads and the number of genera and species that could be identified in total. The family levels of the original PR^2^ taxonomy and the new WoRMS and Algebase were comparable. For species‐level assignment, taxa were disregarded if they did not have full taxonomic information, for example, an assignment to the taxon *Askenasia_sp*. was considered as an assignment only to the genus level.

## 4 Results

### 4.1 Improvements of the Reference Database

The database dedicated to Baltic protists, created with pr2‐wormifier allowed 82,819 sequences to be assigned directly to species level via WoRMS and 23,402 sequences via Algaebase, while taxonomy for 23,515 sequences was inferred from other species within the same genus. An additional 3376 sequences were classified based on genus‐level matches in WoRMS. For 93 sequences, genus‐level searches returned multiple results; in these cases, taxonomy was resolved by comparing entries with PR^2^ or NCBI (Table S1).

**FIGURE 2:**
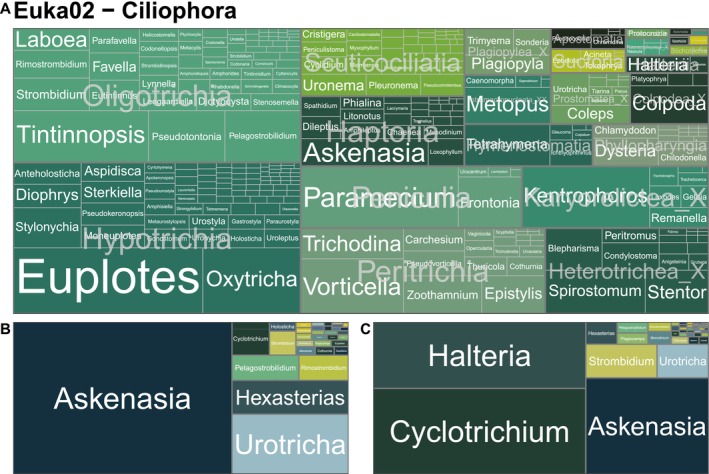
Overview of ciliate sequences of the sedaDNA metabarocding dataset (Euka02) in the new reference database and the resulting species classification. (A) The number of sequences present in the new reference database grouped by class and genus. (B, C) The results of the species identification with the original PR^2^ (v1, B) and the new database (V4, C). The size of the fields represents the relative number of reads assigned to each genus.

These updates resulted in a final database containing 88,682 sequences with species‐level taxonomy and 42,925 with genus‐level taxonomy. Of these, 1812 genera included species known to occur in the Baltic Sea. Overall, the curated database covered 69.7% of all genera expected in the Baltic, an improvement over the 57.6% coverage in the original PR^2^. This also included 64.1% of all ciliate genera, compared to 57.2% previously (Figure 2).

### 4.2 Results of Species Classification

To assess how database improvements affected taxonomic assignment, we compared ciliate and dinoflagellate classifications across four versions of the reference database for both datasets (for more details on the versions, see Comparison of databases and Table 1).

**TABLE 1:** Overview of database curation steps, changes to sequence numbers and the number of unique genera and species of all taxa retained in each database version: V1—original PR^2^ database; V2—cleaned PR^2^ database with PR^2^ taxonomy; V3—cleaned PR^2^ database with WoRMS taxonomy; V4—cleaned PR^2^ database with WoRMS taxonomy with additional NCBI reference sequences. Genus and species numbers represent unique genera and species represented (sp. is not counted as species nor the genus names which refer to a higher taxonomy with a ‘_X’ added). The numbers for database versions 2 and 3 are the same since only the taxonomy of the sequences is changing.

Database curation steps	Database version	Sequence number	Genus	Species
Initial PR^2^ database (v5.1.1)	v1	240,201	25,075	44,022
Removed: non‐18S rRNA sequences	v2	–8832		
Removed: low‐resolution sequences	v2	−60,034		
Removed: missing WoRMS & Algaebase taxonomy	v2	−47,488		
Remaining after filtering	v2/v3	123,847	11,724	25,099
Added: NCBI‐specific sequences	v4	+10,477		+2872
Remaining after updated taxonomy & Adding Species	v4	134,324	12,343	27,971
Removed: *in silico* PCR via CRABS (TAReuk)	Final v4	−37,513		
Removed: *in silico* PCR via CRABS (Euka02)	Final v4	–42,183		
Summary (TAReuk/Euka02)	Final v4	96,811/92,141	10,199/10,954	23,213/23,982

In general, the removal of sequences with low taxonomic resolution from PR^2^ (script 1_Clean_PR2) notably increased both the number of assigned ASVs and the total number of reads to genus and species level (Table 2; Figures S4 and S5). Changing the taxonomy alone had little impact on the results, whereas the addition of sequences from NCBI substantially improved the number of identified taxa. The changes in proportion of reads on the family, genus and species level were not as great for dinoflagellates as for Ciliophora between the different databases.

**TABLE 2:** Overview of ASVs and reads for the TAREuk dataset assigned to genus level, species level, the taxon Ciliophora and Dinophyceae, each assigned to family, genus and species by each version of the database.

TAREuk		General		Ciliate				Dinoflagellates			
Measurement	Db version	Genus	Species	Taxon	Family	Genus	Species	Taxon	Family	Genus	Species
ASVs	v1	9996	5079	2332	1454	951 (29)	332 (16)	3772	1280	978 (34)	390 (39)
	v2	12,226	6129	2318	1670	1273 (44)	203 (26)	4772	1710	1311 (37)	514 (40)
	v3	11,784	6042	2319	1692	1279 (42)	201 (26)	3968	1412	1282 (38)	517 (40)
	v4	11,734	6185	2336	1828	1356 (42)	349 (27)	3864	1211	1135 (40)	493 (41)
Reads in proportion (%)	v1	53.9	30.8	15.9	66.1	43.2	15	11.1	45.5	36.4	6.6
	v2	70.2	36.9	15.9	84.5	65.1	3.2	15.6	49.2	42.2	8.3
	v3	68.4	36.6	15.9	88.6	65.2	3.2	14.3	47.7	45.8	9
	v4	68.5	38.5	15.9	89	65.3	14.1	14.1	47.2	45.5	9.3

*Note:* In brackets, the number of unique genera and species is represented. In brackets, the numbers of unique genera and species are shown. Proportion of reads referred for genus and species in general, as well as the taxon column to the total reads, the taxon species assignments to a certain taxonomic level refer to the total reads of the responding taxon. The total read number was 10,303,999. Database version: 1—original PR^2^ database; 2—cleaned PR^2^ database with PR^2^ taxonomy; 3—cleaned PR^2^ database with WoRMS taxonomy; 4—cleaned PR^2^ database with WoRMS taxonomy with additional NCBI reference sequences.

#### 4.2.1 Ciliophora

During the analysis of the eDNA metabarcoding data (TAREuk), the number of ASVs assigned to ciliates varied between 2332 and 2336 across the four reference databases (Table 2). After removal of sequences with low taxonomic resolution, most newly assigned ASVs belonged to the genus *Strombidium*, with 310,662 additional reads. In contrast, for the genus *Pelagostrobilidium*, the amount of ASVs and reads decreased by 58 ASVs and 4693 reads (Table S7). After adding NCBI sequences, many newly assigned ASVs and reads belonged to the genus *Pelagostrobilidium* (80 ASVs and 5818 reads) and *Kentrophyllum* (24 ASVs and 6188 reads; Figure 2B,C; Table S7). Overall, most reads were assigned to the Baltic genera *Strombidium*, *Rimostrombidium*, and *Pelagostrobilidium* (Figure S1C, Table S7).

Analysing the sedaDNA metabarcoding data (Euka02), the number of ASVs assigned to ciliates varied between 682 and 688 across reference databases (Table 3). After removal of sequences with low taxonomic resolution, most newly assigned ASVs belonged to the genera *Cyclotrichium*, *Askenasia* and *Strombidium*, with additional reads 323,675, 150,269 and 38,816, respectively (Table S10). Adding NCBI sequences led to many newly assigned ASVs and reads belonging to the genus *Halteria* (53 ASVs and 253,460 reads), which had not been detected in any of the earlier database versions (Figure 2B,C, Table S10). Using the reference database created by our pipeline, most reads were assigned to the Baltic genera *Halteria*, *Cyclotrichium* and *Askenasia* (Figure 2C), while most reads were assigned to *Askenasia* with the initial PR^2^ database (Figure 2B; Tables S10–S12).

**TABLE 3:** Overview of ASVs and reads for the Euka02 dataset assigned to genus level, species level, the taxon Ciliophora, ciliate family, ciliate genus and ciliate species by each version of the database.

Euka02		General		Ciliate				Dinoflagellates			
Measurement	Db version	Genus	Species	Taxon	Family	Genus	Species	Taxon	Family	Genus	Species
ASVs	v1	6140	2394	688	141	94 (32)	20 (15)	3805	561	253 (26)	166 (22)
	v2	6616	2411	613	86	232 (36)	30 (18)	4157	792	499 (28)	165 (23)
	v3	6525	2452	625	247	233 (35)	31 (19)	3854	666	496 (28)	164 (23)
	v4	6877	2374	682	321	297 (46)	29 (20)	3796	613	436 (28)	147 (23)
Reads in proportion (%)	v1	47.2	22.6	0.8	13.7	6.8	0.8	22.3	70.9	70.6	69.3
	v2	44.1	21.9	0.8	5.2	28.7	0.8	23.2	71.6	71.3	66.9
	v3	44	21.9	0.8	30.2	28.7	0.8	23	71.9	71.7	67.3
	v4	46.2	23.5	0.8	40.6	39.1	1	23	71.8	71.6	67.2

*Note:* In brackets, the numbers of unique genera and species are shown. Proportion of reads referred for genus and species in general, as well as the taxon column to the total reads, the taxon species assignments to a certain taxonomic level refer to the total reads of the responding taxon. The total read number was 279,749,200. Database version: V1—original PR^2^ database; V2—cleaned PR^2^ database with PR^2^ taxonomy; V3—cleaned PR^2^ database with WoRMS taxonomy; C4—cleaned PR^2^ database with WoRMS taxonomy with additional NCBI reference sequences.

**FIGURE 3:**
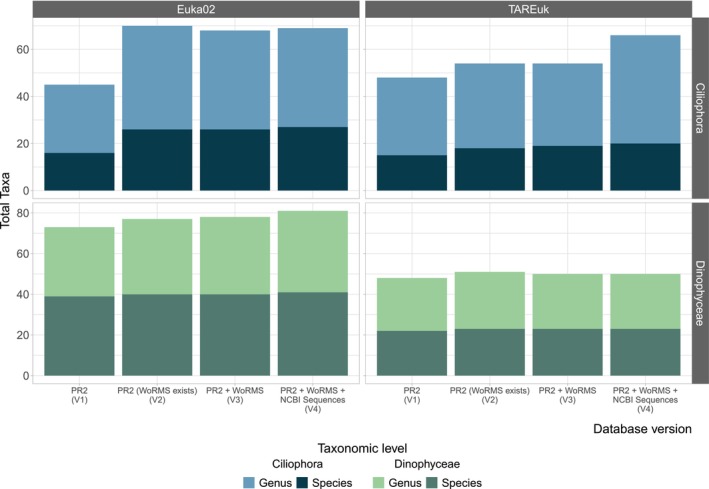
Number of ciliate and dinoflagellate species and genera of both datasets that could be identified by each version of the database.

Applying the pr2‐wormifier resulted in different patterns of change for two datasets. For the TAREuk (eDNA) dataset, the largest shift resulted from complementing the PR^2^ database with GenBank sequences. For Euka02 (sedaDNA), the largest shifts in assigned ASVs, taxa and reads resulted from removing sequences with low taxonomic resolution (Figure 3; Figure S6).

#### 4.2.2 Dinophyceae

In the analysis of the eDNA metabarcoding data (TAREuk), the number of ASVs assigned to dinoflagellates varied between 3772 and 3796 across the four reference databases (Table 2). After removal of sequences with low taxonomic resolution, most newly assigned ASVs belonged to the cosmopolitan genus *Takayama*, with 70,882 additional reads (Table S13), while most newly assigned reads belonged to the Baltic genus *Prorocentrum* with 117,431 additional reads. The addition of NCBI sequences only marginally changed the number of assigned ASVs (Figure S2B,C, Table S13). At the end, most reads were assigned to the Baltic genera *Biecheleria* and *Tripos* (*Ceratium*; Figure S2C, Table S13).

During the analysis of the sedaDNA metabarcoding data (Euka02), the number of ASVs assigned to dinoflagellates varied between 3805 and 3796 across databases (Table 3). Resulting of the removal of sequences, most newly assigned ASVs belonged to the Baltic genera *Gymnodinium* and *Biecheleria*, with 1,771,177 and 315,522 additional reads, respectively (Table S16). After adding NCBI sequences, many newly assigned ASVs and reads belonged to the genera *Gymnodinium* and *Biecheleria*, while the number of reads assigned to these genera decreased. With the final database version, most reads were assigned to the Baltic genus *Biecheleria*, similar to an assignment with the initial PR^2^ database (Figure S3B,C and Tables S16–S18).

In both datasets, assignments of ASVs and reads at the genus and species level increased after sequence removal and decreased again after sequence addition. Overall, the changes were more pronounced in the seawater and surface sediment eDNA dataset (TAREuk) than in the sedaDNA dataset (Euka02).

## 5 Discussion

The pr2‐wormifier pipeline enables researchers to create 18S reference databases that are optimized for their protist species of interest. Keck et al. (2023) described seven possible issues with metabarcoding reference databases: (i) mislabelling of sequences, (ii) sequencing errors, (iii) sequence conflict, (iv) taxonomic conflict, (v) low taxonomic resolution, (vi) missing taxon, (vii) missing intraspecific variants. The highly curated PR^2^ database (Guillou et al. 2013) already controls the first two issues via curating and reassignment of published sequences. It also includes several sequences for one species (the seventh issue). To consider the issues related to incompleteness, pr2‐wormifier adds additional sequences from the more up‐to‐date and extensive NCBI database (Benson et al. 2000). By incorporating WoRMS and AlgaeBase taxonomies into the pipeline, it is able to deal with synonyms of species and genus names and provides a taxonomy that is consistent, comparable to other studies and adaptable to future changes.

### 5.1 Improving the PR^2^ Reference Database

During the benchmarking, two main reasons led to the removal of sequences from the initial PR^2^ database: the sequence either had low taxonomic resolution, or the sequences belonged to a genus that is present neither in WoRMS nor in AlgaeBase. Reducing comprehensiveness by removing sequences from a reference database always bears the risk of creating false positive assignments (Keck et al. 2023). Using the RDP classifier from mothur, false positives are created when a taxon is missing from the database and the sequence is assigned to a related taxon with a sequence more similar to the query than to all others in the database (i.e., having a bootstrap value of 80 or higher, Schloss et al. 2009). Consequently, false positive assignments are less likely when taxa are missing from groups that are well represented in the database, since chances are higher that multiple related taxa exist, whose sequences are equally similar to the one that should be classified. Therefore, having sequences with low taxonomic resolution in the database mainly adds value for taxonomic groups that otherwise would be highly underrepresented.

However, sequences with low taxonomic resolution can obstruct taxonomic classification. When these sequences belong to species that are already present in the database, identification is still only possible at the highest level permitted by the limited resolution. In the PR^2^ database, for example, 209 sequences are identified only to the order Choreotrichida. Even if the species is represented in the database with full taxonomy, such sequences can be identified as Choreotrichida at best. Similar observations were made by Keck et al. (2023), who found that 54% of animal sequences in BOLD are not assigned to the species level and only 82% are assigned to the family level. This low resolution becomes particularly problematic when the amplified sequence is missing from the reference database and the closest matches vary in taxonomic resolution. Since many assignment methods are based on the last common ancestor or phylogenetic placement, incomplete taxonomy can lead to failed assignments or assignments at higher taxonomic ranks. Therefore, keeping sequences with low taxonomic resolution in a database has trade‐offs. In any case, aiming for species or genus‐level assignments requires a comprehensive database. In this context, we think it is reasonable to remove sequences with low taxonomic resolution.

While removing sequences with low taxonomic resolution is crucial for reliable classification, excluding sequences not listed in WoRMS or AlgaeBase enables the switch to a WoRMS‐based taxonomy. This additional filtering step had a limited impact on regional coverage, although 47,488 sequences (~20% of the initial PR^2^ database) were excluded due to missing taxonomy in these sources; only less than 1% of the sequences relevant to the Baltic Sea were affected. The majority of removed sequences represent non‐marine taxa, for example, 25% belonging to Hexapoda and 37% to Fungi, which fall outside the scope of WoRMS and AlgaeBase. According to Mugnai et al. (2023), marine datasets, after excluding terrestrial taxa such as insects, showed improved classification accuracy, with minimal negative effects on assignments at higher taxonomic levels. To ensure the detection of potential contaminants or benthic species, we retained terrestrial taxa present in WoRMS by disabling its marine‐only filter. Overall, this strategy maintained sensitivity to the diverse range of taxa present in our study environments while the use of a WoRMS‐based taxonomy enhanced the compatibility of the PR^2^ database with other sources of reference sequences as well as the comparability of results.

### 5.2 Assignment of the TAREuk and Euka02 Datasets

In the example of ciliates as well as dinoflagellates (Dinophyceae), it became evident that both the removal of sequences with low taxonomic resolution and the addition of available sequences from NCBI to the reference database have an impact on the taxonomic classification: both increased the number of ASVs that could be classified to genus or species level, similarly to the total number of species and genera that could be identified. It has previously been observed that adding sequences from non‐represented species improves assignment resolution (Fueyo et al. 2024; Mugnai et al. 2023). The problems with low taxonomic resolution are also well known. Sequences in reference databases annotated only to broad ranks such as class or phylum prevent assignment methods from providing finer resolution when these serve as the nearest reference simultaneously with sequences assigned to species or genus level. This makes fine‐scale taxonomic resolution impossible, a problem particularly pronounced within taxonomically more challenging organisms (Keck et al. 2023; Liu and Zhang 2021; Somervuo et al. 2017).

Due to incomplete taxonomy, 6647 sequences for ciliates and 3882 sequences of dinoflagellates were removed from the newly generated database. The majority of the assigned genera for ciliates are listed by Telesh et al. (2008) as occurring in the Baltic Sea and were expected in our dataset, which supports the reliability of these assignments rather than suggesting they are false positives (> 90% of all ASVs). The proportion of Baltic genera in dinoflagellates is lower compared to ciliates. This is likely an artefact of the reference taxonomic checklist (HELCOM 2004), probably resulting from its age. As an example, two genera detected in our dataset, *Biecheleria* and *Apocalathium*, were only formally described for the Baltic Sea in 2005 and 2009, respectively, having previously been placed within *Scrippsiella* (Kremp et al. 2005; Sundström et al. 2009). Their absence from the Baltic checklist thus reflects missing taxonomic information rather than a true distributional signal. The same might be happening also in the case of other genera. Nonetheless, removing sequences with low taxonomic resolution might not always be suitable for the best possible identification of other taxonomic groups, for example, the parasitic Syndiniales, where very few sequences exist with taxonomic information to genus or species level. Changing from the PR^2^ taxonomy to WoRMS and AlgaeBase had only a small impact on the taxonomic resolution at the genus and species level. This is not surprising, since the taxonomic levels of species within the same genus should share the same upstream taxonomy (Keck et al. 2023). The increase in family‐level assignments may be linked to gaps in the original PR^2^ database, where family information was missing for sequences with a known genus. For example, the genus *Castula* was assigned only the order name Armophorea in place of a family name. These problems were also already observed by for example Liu and Zhang (2021).

Comparing the two datasets across both taxonomic groups reveals notable differences in the extent of taxonomic assignment improvement. For the seawater and surface sediment eDNA metabarcoding dataset, genus‐level assignment improved substantially for both taxa (> 20% for ciliates and ~10% for dinoflagellates). In contrast, for the sedaDNA metabarcoding dataset, only ciliate assignment improved greatly (> 30%), while dinoflagellate assignment remained largely unchanged (~1% increase at genus level). This discrepancy likely reflects the influence of community composition on the effectiveness of reference database improvements.

### 5.3 Limitations & Recommendations

PR^2^‐wormifier can only be used to create reference databases for 18S sequences of eukaryotes since it is based on the PR^2^ database, which contains only very few sequences from other domains. Additionally, it is limited to marine communities since it relies on WoRMS and AlgaeBase, which mostly contain marine species. While the pipeline aims to find all relevant sequences, the comprehensiveness of the resulting database is limited by the availability of sequences in public databases. Therefore, the user should always check if the resulting database is extensive enough for taxonomic assignments at the desired level.

Furthermore, not all sequences in the created database cover the full length of the 18S rRNA gene. Depending on the used classification method, sequences that do not cover the amplified region might influence the results. NCBI might also contain sequences that are in reverse complement. Therefore, we recommend using *in silico* PCR (e.g., as implemented in CRABS; Jeunen et al. 2023) to reduce the database to full‐length sequences of the region that is amplified by the barcoding primer before taxonomic classification.

Users should also be aware that while our pipeline corrects taxonomy and resolves synonyms for sequences added from NCBI, it relies on the provided species or genus identification to be correct with no possibility of identifying misclassified sequences. However, a study by Leray et al. (2019) found that major taxonomic errors in GenBank are rarer than often assumed, with error rates of approximately 0.01% at the class level and likely below 1% even at the genus level, suggesting the practical impact of this limitation may be modest. Nonetheless, adding sequences from NCBI bears the risk of adding sequences that have been intentionally not included in the PR^2^ database as part of its curation process. We see the strength of that step of the pipeline in being able to add sequences that have been published past the last release of the PR^2^ database as well as sequences that are out of the scope for PR^2^, but might be of interest to the researcher e. g. when using universal primers that do not only amplify protist sequences. The metadata file provides information to the user from which source sequences were added. Through the provided species list the user can control for which taxa sequences should be added. By modifying the search query it is also possible to limit the scope of the NCBI search e. g. to sequences published after a certain date. The extent to which sequences are added from NCBI presents a trade‐off between the level of curation and comprehensiveness of the resulting database.

A challenge when creating the new taxonomy is the existence of homonyms, especially at the genus level (Keck et al. 2023). We aimed to resolve this issue by comparing the WoRMS or AlgaeBase taxonomy to the original NCBI or PR^2^ taxonomy of a sequence to avoid allocating a wrong taxonomy from a different genus with an identical name. Since NCBI, PR^2^, WoRMS, and AlgaeBase are not identical in the ranks and taxa names they use, this process is not perfect. Therefore, it is possible that in rare cases a wrong taxonomy is allocated.

Finally, uncertainties inherently remain for sequences that are only assigned to genus level, such as *Biecheleria* sp. While taxonomy updating is performed based on the genus name, this approach cannot account for cases where a species has been transferred to a different genus, as discussed for the *Scrippsiella* complex. Since the species identity of such sequences is unknown, potential genus‐level reclassifications will not be reflected in the updated taxonomy and these sequences may therefore retain an outdated genus assignment. In the long term, such sequences can only be reliably resolved through taxonomic expert reidentification of the underlying organisms (Keck et al. 2023). Ultimately, the automated validation implemented in our pipeline is not intended to replace the expert curation that initiatives like PR^2^ provide, but rather serves as a practical workaround to bridge the gap until globally curated databases achieve broader taxonomic coverage.

## Author Contributions

Stefanie Knell and Juliane Romahn conceived and designed the project. Stefanie Knell and Juliane Romahn wrote the scripts. Stefanie Knell analysed data and wrote the first draft of the paper. Juliane Romahn and Miklós Bálint supervised the project. Miklós Bálint provided conceptual guidance and acquired the funding. All authors edited, read and approved the final manuscript.

## Funding

The software was developed as part of the PHYTOARK project which was funded by the K314/2020 grant of the Leibniz Collaborative Excellence program. Miklós Bálint was supported by the LOEWE Centre for Translational Biodiversity Genomics, funded by the Hessen State Ministry of Higher Education, Research and the Arts (HMWK) through project LOEWE/1/10/519/03/03.001(0014)/52. Miklós Bálint received support from the Leibniz ScienceCampus ‘Geogenomic Archaeology Campus Tübingen (GACT)’ (project number W73/2022) supported by the Leibniz Association, the Ministry of Science, Research and the Arts of Baden‐Württemberg and the University of Tübingen.

## Conflicts of Interest

The authors declare no conflicts of interest.

## Supporting information


**Table S1:** Detailed overview of the number of Taxa and Sequences present in the different databases.
**Table S2:** Overview of the amount of the higher taxa that are removed from the original PR2 database.
**Table S3:** Overview of removed ciliate sequences and the families they belong to.
**Table S4:** Overview of removed Dinophyceae sequences and the families they belong to.
**Table S5:** Overview of the amount of sequences and ASVs that were assigned by the different strategies.
**Table S6:** Various statistics on the amount of taxa and sequences of the species list, database V1 and V4.

**Table S7:** Comparison of the amount of ASVs and reads of the TAREuk dataset assigned to a certain genera for each database version and the difference compared to the previous database version.
**Table S8:** Taxonomic assignment of Ciliophora for the TAREuk dataset at the family level, bootstrap values and read counts for each ASV across database versions.
**Table S9:** Taxonomic assignment of Ciliophora for the TAREuk dataset at the species level, bootstrap values and read counts for each ASV across database versions.
**Table S10:** Comparison of the amount of ASVs and reads of the Euka02 dataset assigned to a certain genera for each database version and the difference compared to previous database version.
**Table S11:** Taxonomic assignment of Ciliophora for the Euka02 dataset at the family level, bootstrap values and read counts for each ASV across database versions.
**Table S12:** Taxonomic assignment of Ciliophora for the Euka02 dataset at the species level, bootstrap values and read counts for each ASV across database versions.

**Table S13:** Comparison of the amount of ASVs and reads of the TAREuk dataset assigned to a certain genera for each database version and the difference compared to the previous database version.
**Table S14:** Taxonomic assignment of Dinophyceae for the TAREuk dataset at the family level, bootstrap values and read counts for each ASV across database versions.
**Table S15:** Taxonomic assignment of Dinophyceae for the TAREuk dataset at the species level, bootstrap values and read counts for each ASV across database versions.
**Table S16:** Comparison of the amount of ASVs and reads of the Euka02 dataset assigned to a certain genera for each database version and the difference compared to previous database version.
**Table S17:** Taxonomic assignment of Dinophyceae for the Euka02 datasetat the family level, bootstrap values and read counts for each ASV across database versions.
**Table S18:** Taxonomic assignment of Dinophyceae for the Euka02 dataset at the species level, bootstrap values and read counts for each ASV across database versions.

**Figure S1:** Overview of ciliate sequences of the environmental DNA metabarcoding dataset (TAREuk).
**Figure S2:** Overview of dinoflagellate sequences of the environmental DNA metabarcoding dataset (TAREuk).
**Figure S3:** Overview of dinoflagellate sequences of the sedaDNA metabarcoding dataset (Euka02).
**Figure S4:** Number of ASVs that could be identified to species and genus level.
**Figure S5:** Number of reads that could be identified to species and genus level.
**Figure S6:** Change in bootstrap support values within the mothur assignments at the genus level.

## Data Availability

The bioinformatics pipeline is accessible via GitHub: https://github.com/jromahn/PR2‐wormifier. The created reference databases for the Baltic Sea are accessible via FigShare at the following DOIs: Baltic Sea protist reference database—10.6084/m9.figshare.30225976; Baltic Sea protist reference database subset for the TAREuk—10.6084/m9.figshare.32253564, Baltic Sea protist reference database subset for the Euka02–10.6084/m9.figshare.30225949.
