# Supplementary material for: pr2‐Wormifier: A Bioinformatics Pipeline to Create Custom Reference Databases for Improved Metabarcoding of Marine Protists
Source: Mol Ecol Resour. 2026 Jun 23;26(5):e70168. doi: 10.1111/1755-0998.70168 (PMC13288752; doi:10.1111/1755-0998.70168)
Supplement: Supplementary file 4 — Figure S1: Overview of ciliate sequences of the environmental DNA metabarcoding dataset (TAREuk). Figure S2: Overview of dinoflagellate sequences of the environmental DNA metabarcoding dataset (TAREuk). Figure S3: Overview of dinoflagellate sequences of the sedaDNA metabarcoding dataset (Euka02). Figure S4: Number of ASVs that could be identified to species and genus level. Figure S5: Number of reads that could be identified to species and genus level. Figure S6: Change in bootstrap support values within the mothur assignments at the genus level. [file MEN-26-e70168-s003.docx]

pr2-wormifier:
A bioinformatics pipeline to create custom reference databases for improved metabarcoding of marine protists

Supplementary Figures

**Authors:** Stefanie Knell*^1,2^, Juliane Romahn* ^1,2,3^, Miklós Balint ^1,2,3^

**Corresponding author:** Juliane Romahn [juliane.romahn@stud.uni-giessen.de](mailto:juliane.romahn@stud.uni-giessen.de)

**Keywords:** environmental DNA, taxonomic affiliation, 18S rRNA, WoRMs, sedaDNA, ciliate

- * - both authors contributed equally to the research

**Affiliations:**

1. Justus Liebig University Giessen, Giessen, Germany

2. Senckenberg Biodiversity and Climate Research Centre, Frankfurt am Main, Germany

3. Centre for Translational Biodiversity Genomics, Frankfurt am Main, Germany

# Structure

**Representation of genera** (as tree maps, as in Figure 2)

- **Fig. S1 - Overview of ciliate sequences of the environmental DNA metabarcoding dataset (TAREuk)**
- **Fig. S2 - Overview of dinoflagellate sequences of the environmental DNA metabarcoding dataset (TAREuk)**
- **Fig. S3 - Overview of dinoflagellate sequences of the sedaDNA metabarcoding dataset (Euka02)**

**Assignments to Genus and Species** (for ASV and read number as in Figure 3 for taxa)

- **Fig. S4 - Number of ASVs that could be identified to species and genus level**
- **Fig. S5 - Number of reads that could be identified to species and genus level**

**Changes in bootstrap values**

- **Fig S6 - Change in bootstrap support values within the mothur assignments at the genus level**

# Representation of genera


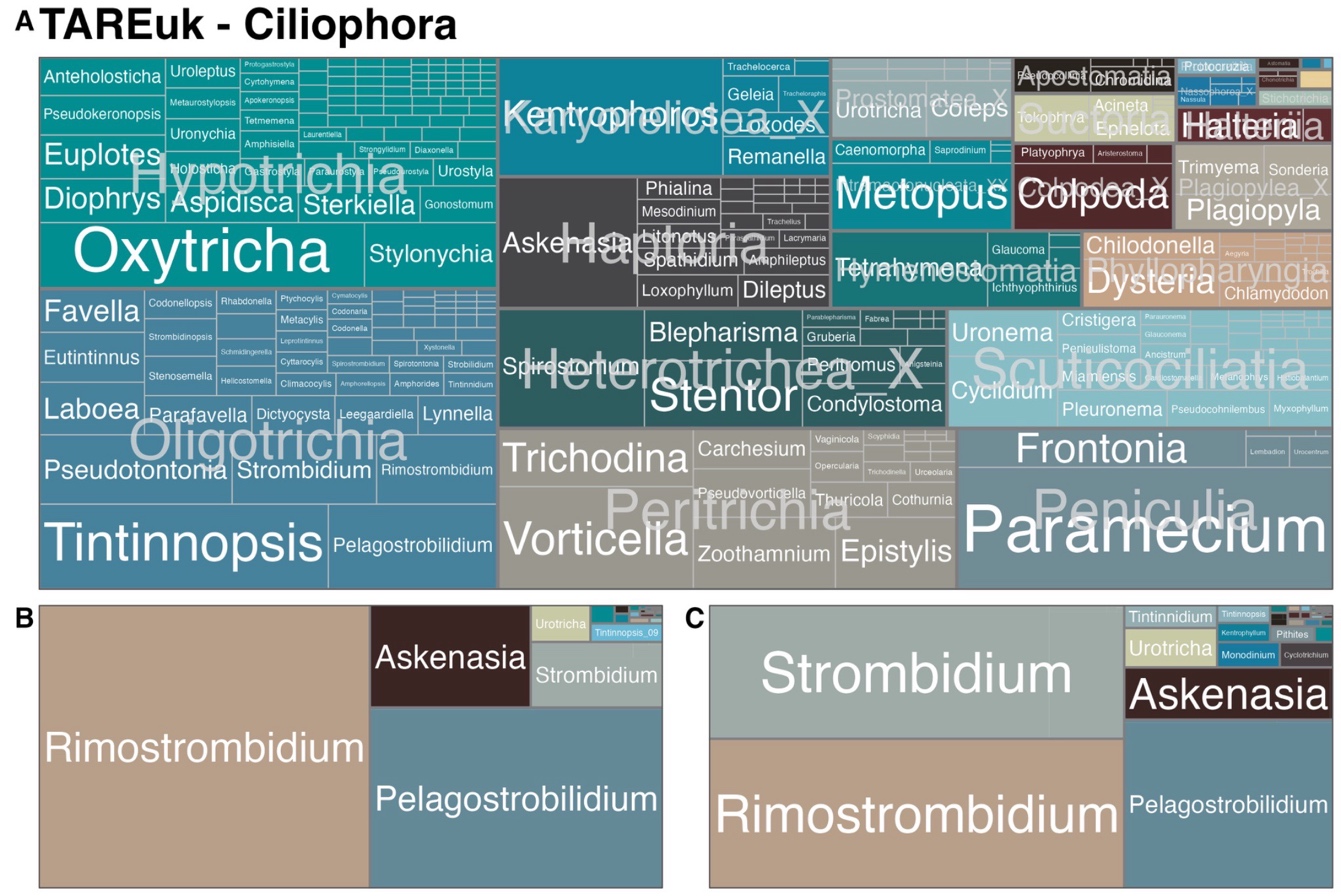


**Fig. S1. Overview of ciliate sequences of the environmental DNA metabarcoding dataset (TAREuk)** in the new reference database and the resulting species classification. A) The number of sequences present in the new reference database grouped by class and genus. B & C) The results of the species identification with the original PR² (v1, B) and the new database (V4, C). The size of the fields represents the relative number of reads assigned to each genus.


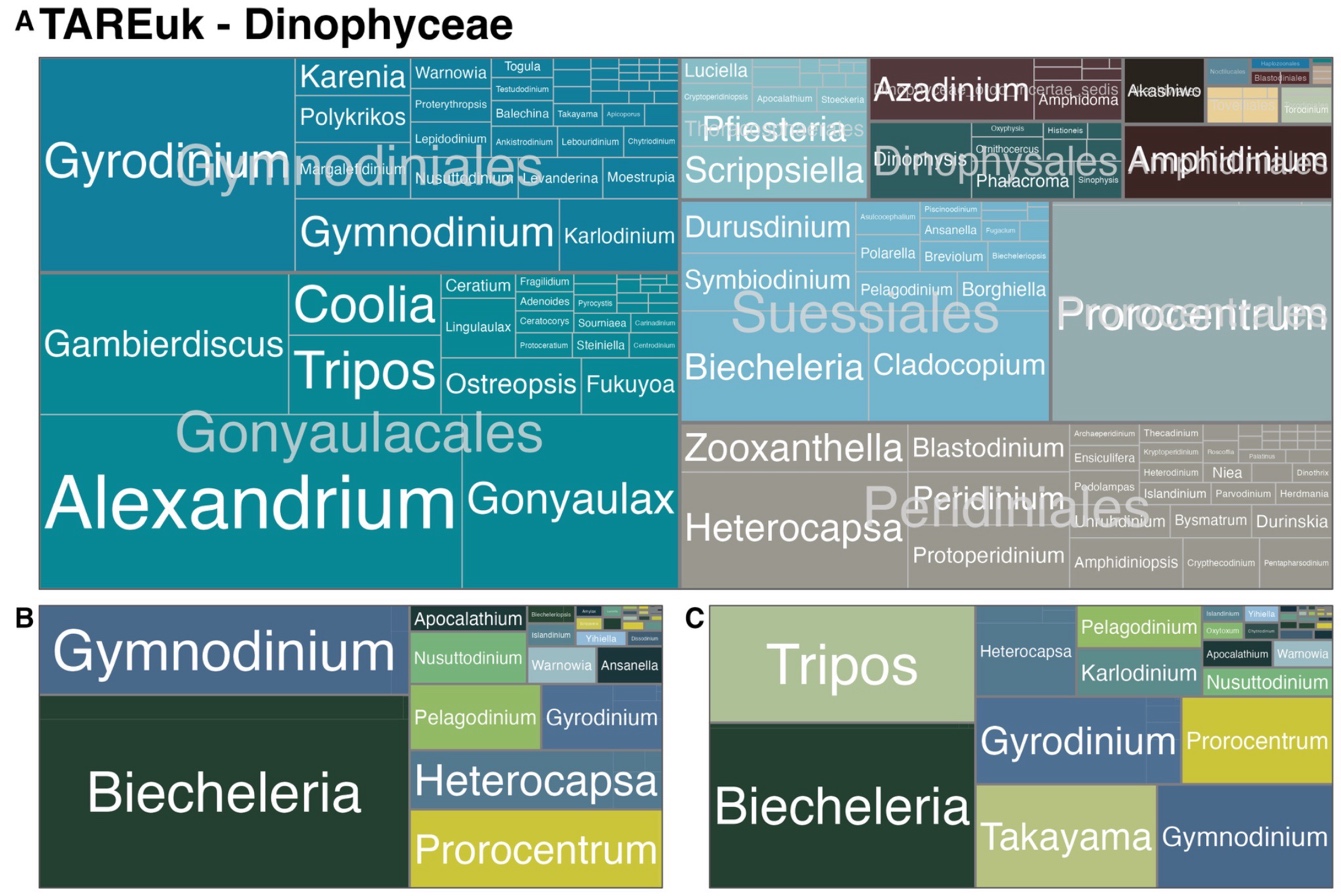


**Fig. S2. Overview of dinoflagellate sequences of the environmental DNA metabarcoding dataset (TAREuk)** in the new reference database and the resulting species classification. A) The number of sequences present in the new reference database grouped by class and genus. B & C) The results of the species identification with the original PR² (v1, B) and the new database (V4, C). The size of the fields represents the relative number of reads assigned to each genus.


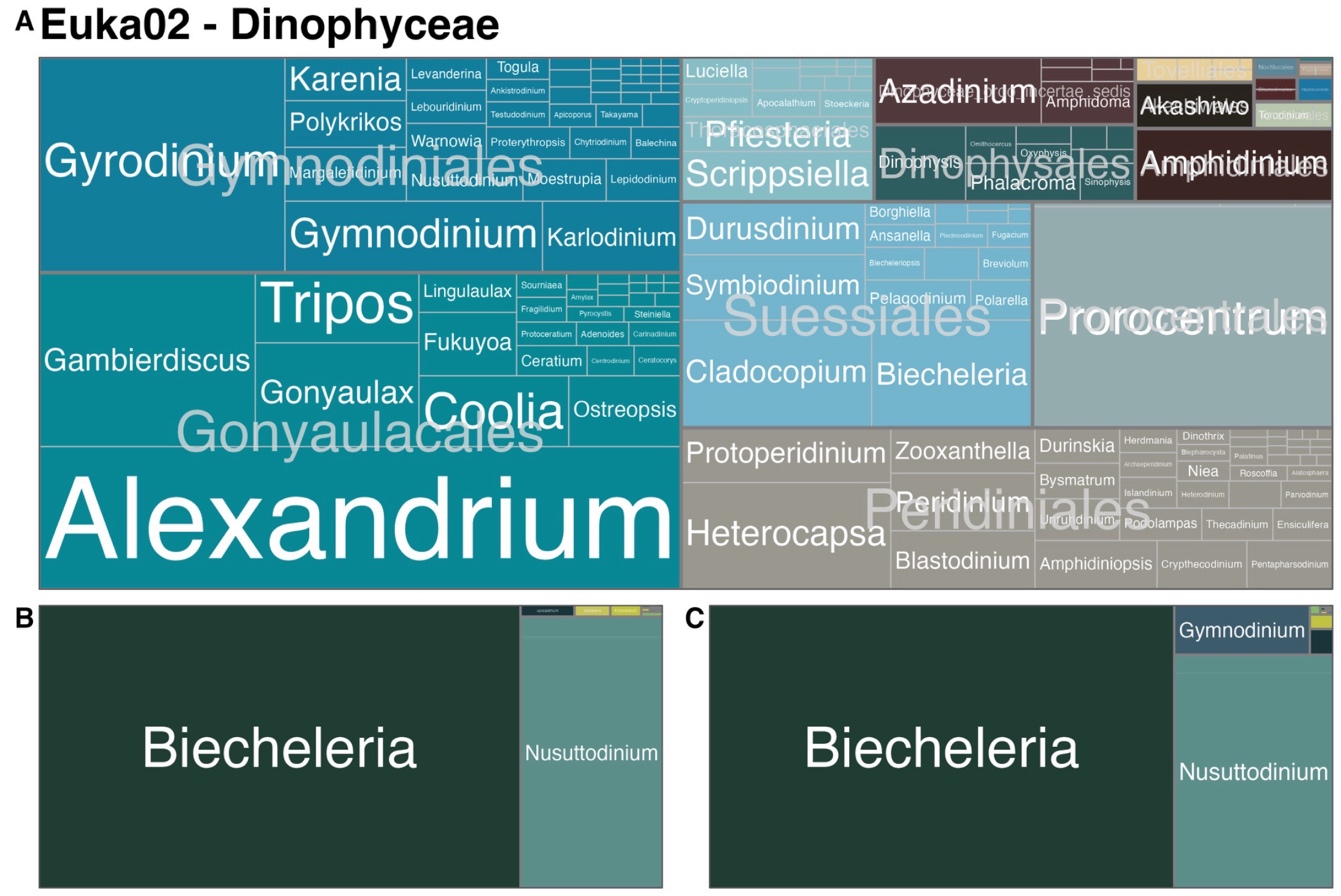


**Fig. S3. Overview of dinoflagellate sequences of the sedaDNA metabarcoding dataset (Euka02)** in the new reference database and the resulting species classification. A) The number of sequences present in the new reference database grouped by class and genus. B & C) The results of the species identification with the original PR² (v1, B) and the new database (V4, C). The size of the fields represents the relative number of reads assigned to each genus.

## Assignments to Genus and Species


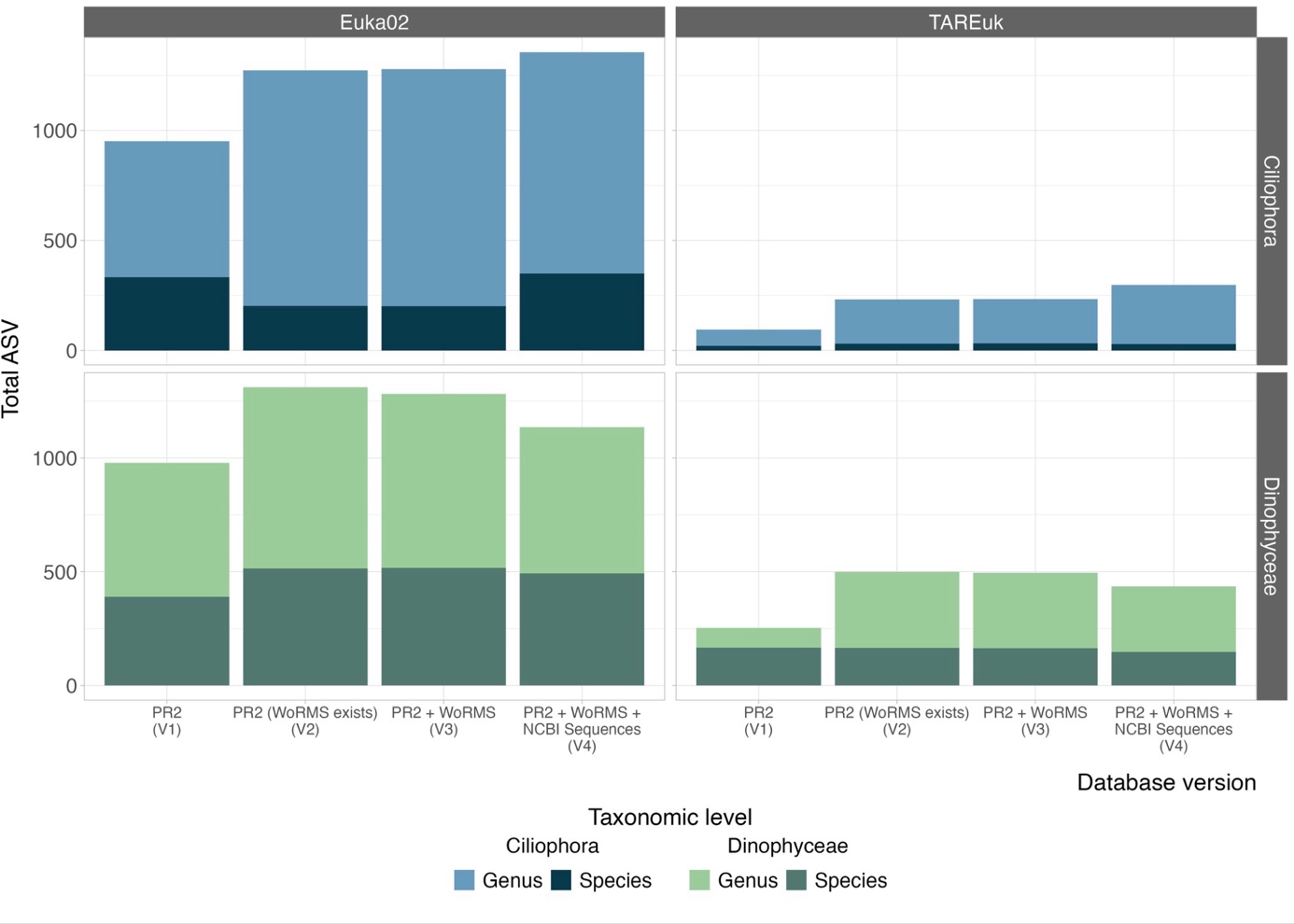


**Fig. S4. Number of ASVs that could be identified to species and genus level** by each version of the pipeline for both taxonomic groups and both datasets.


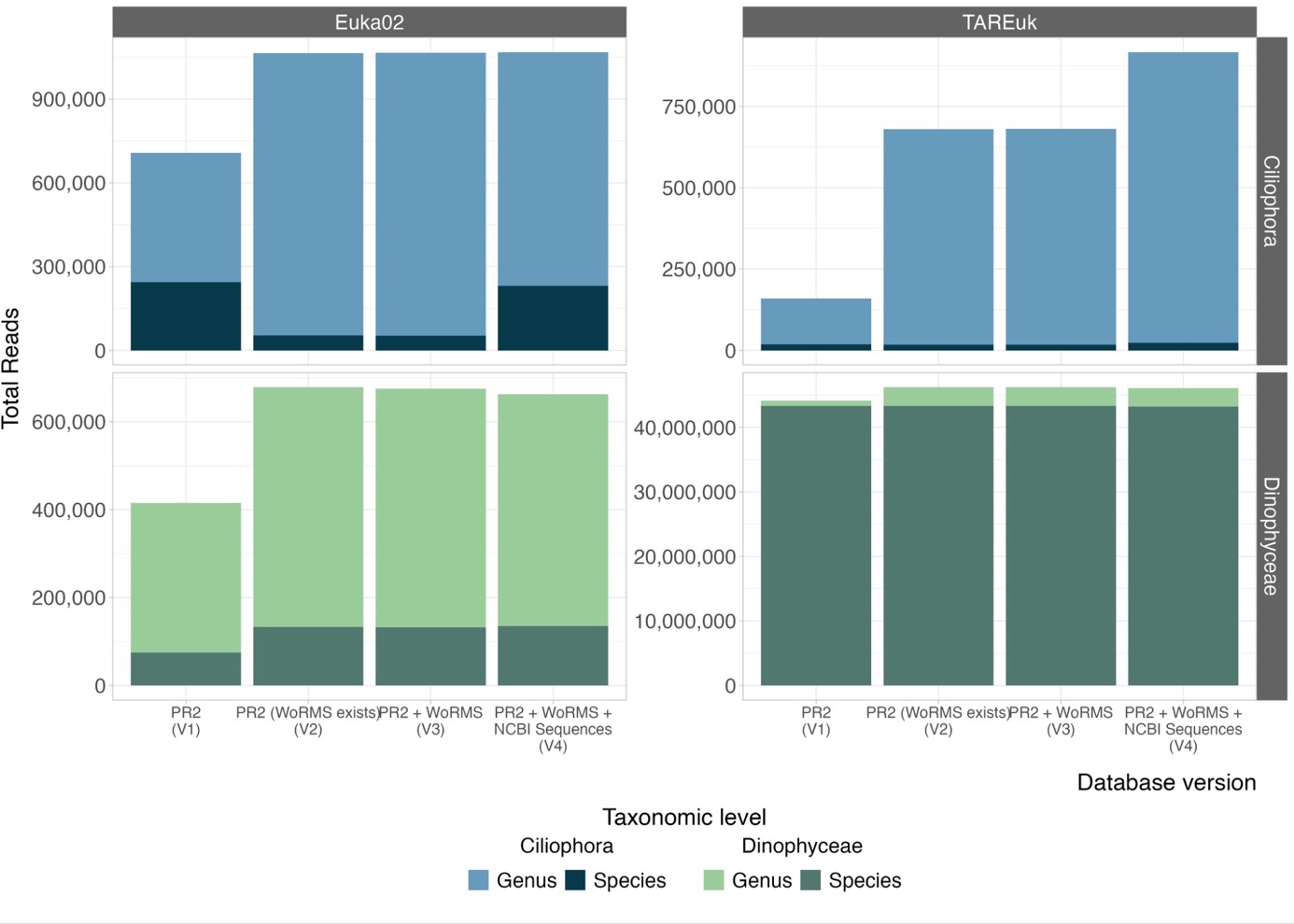


**Fig. S5. Number of reads that could be identified to species and genus level** by each version of the pipeline for both taxonomic groups and both datasets.

## Changes in bootstrap values


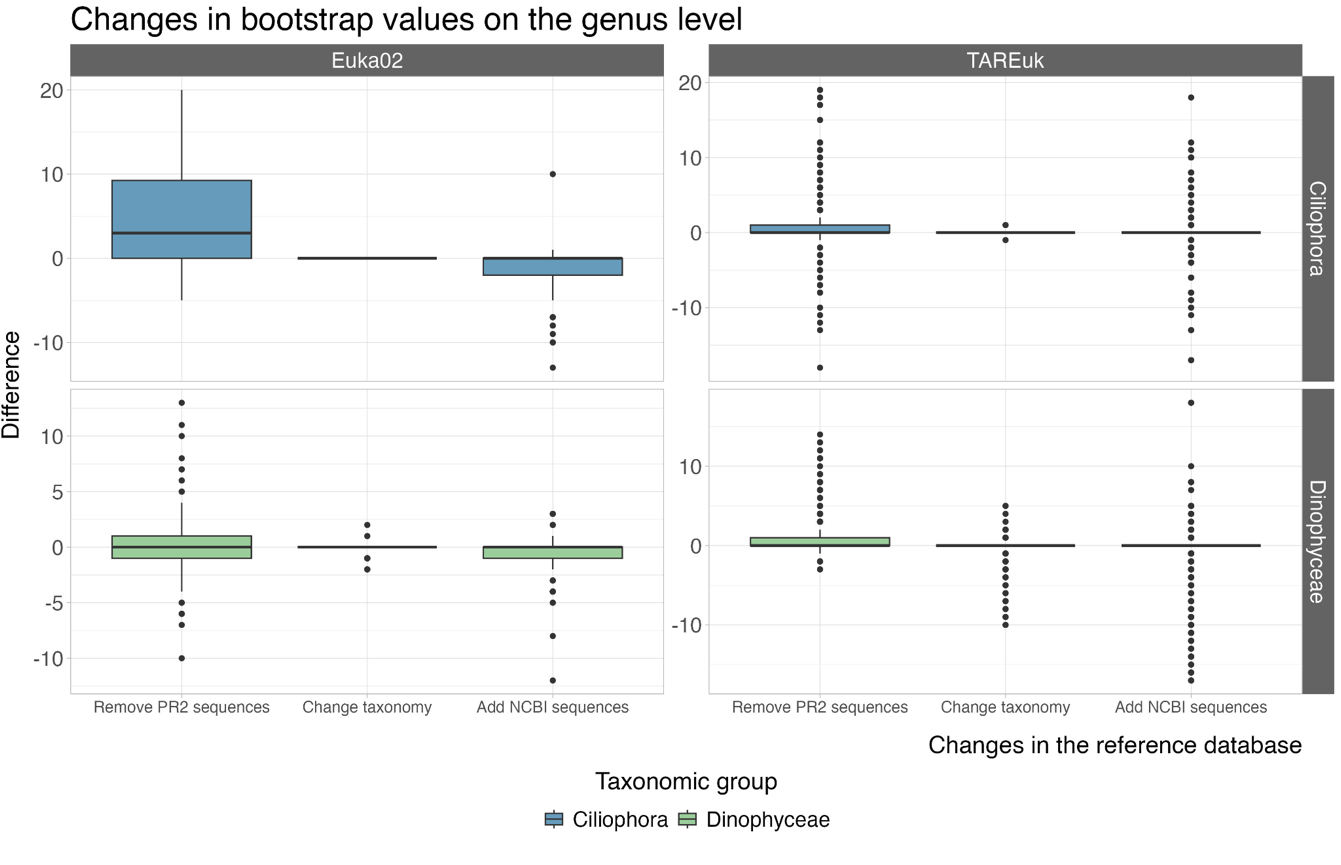


**Fig S6 . Change in bootstrap support values within the mothur assignments at genus level** compared to the changes to the previous version of the database for both taxonomic groups and both datasets.
